# Immune-coagulation dynamics in severe COVID-19 revealed by autoantibody profiling and multi-omics integration

**DOI:** 10.1038/s41598-025-17054-6

**Published:** 2025-09-01

**Authors:** Anoop T. Ambikan, Axel Cederholm, Sefanit Rezene, Maribel Aranda-Guillén, Hampus Nordqvist, Carl Johan Treutiger, Ronaldo Lira-Junior, Nils Landegren, Soham Gupta

**Affiliations:** 1 The Systems Virology Laboratory, Division of Clinical Microbiology, Department of Laboratory Medicine, Karolinska Institutet, 141 52 Stockholm, Sweden; 2 Science for Life Laboratory, Department of Medical Biochemistry and Microbiology, Uppsala university, Uppsala, Sweden; 3 Centre for Molecular Medicine, Department of Medicine (Solna), Karolinska Institutet, Stockholm, Sweden; 4 Department of Infectious Diseases/Venhälsan, South General Hospital, Stockholm, Sweden; 5 Department of Medicine Huddinge, Division of Infectious Diseases, Karolinska Institutet, I73, Karolinska University Hospital, Huddinge, 141 86 Stockholm, Sweden; 6 Division of Oral diagnostics & Surgery, Department of Dental Medicine, Karolinska Institutet, Huddinge, Sweden

**Keywords:** Autoantibodies, Coagulation gene expression, Severe COVID-19, Immune-coagulation dynamics, Thromboinflammatory diseases, Coagulation system, Infectious diseases

## Abstract

Severe COVID-19 is characterized by immune-coagulation dysregulation, yet the contribution of related autoantibodies remains poorly understood. We investigated relationships between plasma autoantibody reactivities, whole-blood transcriptomics, plasma proteomics, and clinical laboratory parameters in a cohort of hospitalized COVID-19 patients. Transcriptomic analysis revealed that 42 curated coagulation and complement cascade genes were upregulated in severe cases compared to healthy controls, with 15 genes, including CR1L, ELANE, ITGA2B, ITGB3, VWF, TFPI, PROS1, MMRN1, and SELP (> 1.2 log2 fold-change), also significantly different from mild cases. Autoantibody profiling against eight coagulation-related proteins (ADAMTS13, Factor V, Protein S, SERPINC1, Apo-H, PROC1, Prothrombin, and PF4) showed reactivities below positivity thresholds across all groups. Using an exploratory approach, in severe cases, subthreshold autoantibody candidates (FDR < 0.25) showed negative correlation trends with select gene expressions and inflammatory markers (Factor V with IL-6 and CXCL10), suggesting potential disease-specific immunomodulatory associations. In contrast, while mild cases exhibited stronger gene-protein correlations, they showed limited associations with antigen reactivities or clinical laboratory parameters. Additionally, no correlations were observed between autoantibodies and platelet-counts or Fibrin-D-dimer levels. Age-associated increases in antigen reactivities were noted in severe disease, implying a role for immunosenescence. These findings support further investigation into the role of subthreshold autoantibody candidates in thromboinflammatory COVID-19 pathogenesis.

**Supplementary Information:**

The online version contains supplementary material available at 10.1038/s41598-025-17054-6.

## Introduction

Coronavirus disease 2019 (COVID-19) is not only a respiratory illness but also a systemic condition in some instances characterized by significant immune dysregulation and hypercoagulability, particularly in severe cases. The severe manifestations of COVID-19 are occasionally driven by an excessive immune response coupled with a hypercoagulable state, both triggered by SARS-CoV-2 infection. This hypercoagulable state, characterized by coagulopathy and thrombosis, contributes to systemic microangiopathy, thromboembolism, and ultimately multi-organ failure^1,2^.

The interplay between inflammation and coagulation in COVID-19 is a key factor in disease severity. SARS-CoV-2 infection occasionally induces a robust inflammatory response in some patients, often described as a cytokine storm, which includes elevated levels of IL-6, TNF-α, and IL-1β. These cytokines activate the coagulation cascade, particularly the extrinsic pathway through tissue factor expression on endothelial cells and monocytes, leading to fibrin formation^3^. Simultaneously, the inflammatory response suppresses natural anticoagulant mechanisms, including antithrombin (SERPINC1) and the protein C system (Factor V and Protein S), further promoting a prothrombotic state^4^.

This bidirectional relationship between inflammation and coagulation potentially creates a vicious cycle that exacerbates disease severity. For instance, thrombin (prothrombin), beyond its role in coagulation, also activates protease-activated receptors (PARs) on endothelial cells and platelets, further fueling inflammation^5^. The interplay between immune, complement, and coagulation systems is a critical factor in some adverse outcomes of SARS-CoV-2 infection, contributing to both inflammation and thrombosis^6^.

Emerging evidence suggests that autoantibodies, particularly those targeting type-I interferons and other molecules, play a significant role in COVID-19 and are associated with adverse clinical outcomes^7–9^. Specifically, Zuo et al. (2020) reported that approximately 50% of serum samples from COVID-19 patients were positive for antiphospholipid autoantibodies^9^. Specific autoantibodies, such as those targeting prothrombin, have been detected following SARS-CoV-2 infection and are influenced by the strength of the antibody response against viral proteins, further implicating their role in COVID-19 severity^10^. Furthermore, injecting IgG fractions from these patients into mouse models resulted in enhanced venous thrombosis, highlighting the complex interplay between autoimmunity, inflammation, and coagulation in COVID-19^9^.

Given this intricate relationship, it is crucial to investigate how autoantibodies targeting coagulation-related factors may influence gene expression profiles, particularly in severe COVID-19. Autoantibodies such as those against ADAMTS13, Factor V, Protein S, SERPINC1, Apo-H, PROC1, and prothrombin could be hypothesized to exacerbate the dysregulation of the coagulation system, further intensifying the inflammatory response and leading to worse clinical outcomes. For example, autoantibodies against ADAMTS13, which regulates von Willebrand factor^11^, can impair its normal functions, thereby increasing thrombotic risk as seen in COVID-19 patients who exhibit a higher prevalence of ADAMTS13 antibodies and markedly reduced ADAMTS13 activity compared to healthy individuals^12^.

Despite growing evidence of the role of autoantibodies in COVID-19, a critical gap remains in understanding their correlation with disease severity and coagulation abnormalities. Transcriptomic analyses have identified distinct gene expression signatures linked to COVID-19 severity, and considering the presence of specific autoantibodies may help to elucidate this^13,14^. Furthermore, the integration of multi-omics layers including transcriptomics, plasma proteomics, and clinical laboratory parameters offers an opportunity to uncover coordinated or disrupted molecular signatures associated with autoantibody profiles. To bridge these knowledge gaps, this study sets out to explore the relationship between autoantibodies targeting coagulation-related factors and COVID-19 severity by integrating autoantibody profiling, transcriptomic expression, and systemic proteomic data with relevant clinical inflammation and coagulation markers. By leveraging quantitative correlation analyses across these molecular and clinical layers, our aim is to uncover novel insights into how gene expressions and subthreshold autoantibody reactivities may influence COVID-19 pathogenesis, thereby informing future investigations into potential biomarkers and new therapeutic targets.

## Results and discussion

### Clinical indicators and transcriptomic changes in coagulation and complement cascades in COVID-19 patients

**Fig. 1:**
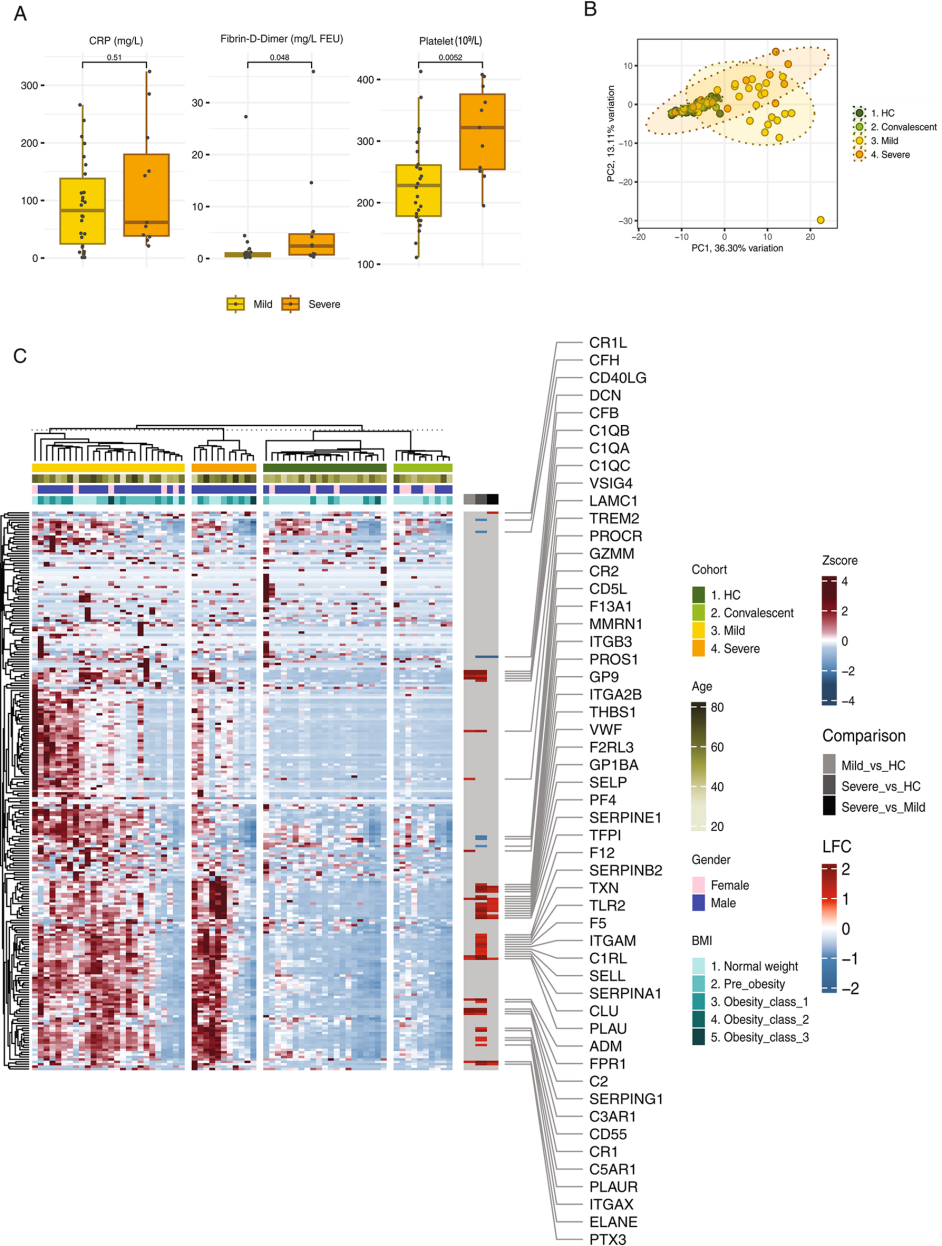
Clinical and transcriptomic profiling of complement-coagulation related pathways in COVID-19 and control groups. **(A) **Dot plots showing clinical laboratory measurements of inflammation (CRP), coagulation (Fibrin-D-dimer), and platelet count in patients with mild (*n* = 26) and severe (*n* = 11) COVID-19. Statistical significance was assessed using Mann-Whitney U tests, with p-values indicated for each comparison. A p-value of <0.05 was considered statistically significant. While CRP levels were not significantly different (*p* = 0.51), both D-dimer and platelet counts were significantly elevated in severe cases (*p* = 0.048 and *p* = 0.0052, respectively), indicating enhanced thromboinflammatory activity. **(B)** Principal Component Analysis (PCA) displaying the sample distribution based on the expression data of 233 curated genes within the ‘Complement-Coagulation related pathways’ across 21 COVID-19-negative controls (HC), 10 convalescent, 26 mild, and 11 severe COVID-19 cases. The PCA illustrates disease-severity-associated separation, with PC1 and PC2 explaining 36.3% and 13.11% of the total variance, respectively. **(C)** Heatmap displaying z-score normalized expression of selected coagulation and complement-related genes across patient groups, stratified by demographic variables (age, gender, BMI). Color intensity of the heatmap encodes z-score normalized expression levels, accentuating marked upregulation in severe cases compared to others. Comparative analysis includes mild vs. HC, severe vs. HC, and severe vs. mild, with expression fold changes annotated. Comparative log2 fold-change values (LFC) for mild vs. HC, severe vs. HC, and severe vs. mild are shown. Statistical significance was assessed using DESeq2 in R, with p-values adjusted for multiple comparisons using the Benjamini-Hochberg adjustment.

To begin our investigation into immune-coagulation changes in COVID-19, we first assessed the standard clinical markers of inflammation (CRP) and coagulation (Platelet counts and Fibrin-D-dimer) using available clinical laboratory parameters from our patient cohort, which included 21 COVID-19-negative controls (HC), 10 convalescent, 26 patients with mild disease and 11 with severe disease described in our earlier study^15^. Fibrin-D-dimer and platelet counts were significantly elevated in patients with severe COVID-19 compared to mild cases (*p = 0.048* and *0.005*, respectively), while CRP levels showed no significant difference (*p = 0.51*) (Fig. 1A). These clinical laboratory parameters reflect downstream coagulation and inflammatory processes and prompted further molecular analysis to identify the upstream regulatory signatures involved.

We subsequently performed transcriptomic profiling to explore upstream molecular alterations linked to thromboinflammation in COVID-19, reanalyzing data from our previous study^15^. To comprehensively capture coagulation and complement dysregulation, we curated a gene set by integrating components from multiple pathway databases, including KEGG, Reactome, WikiPathways, MSigDB, Panther and Gene Ontology (see Supplementary Table S1). Analysis was performed on curated list of 233 genes. Principal Component Analysis (PCA) of significantly altered complement and coagulation-related genes revealed clear separation between clinical groups (Fig. 1B). Severe cases and healthy controls formed distinct clusters, while mild and convalescent individuals showed intermediate or overlapping profiles. This distribution reflects a progressive transcriptomic shift in coagulation pathways with disease severity and recovery, supporting the context-specific regulation of these genes during COVID-19. The heatmap of z-scaled expression shows that gene expression levels were elevated in COVID-19 patients, with a particularly pronounced increase observed in those with severe disease. As shown in Fig. 1C, 15 genes related to the complement-coagulation cascade, were significantly upregulated and DCN1 to be downregulated in severe disease as compared to mild disease state (Supplementary Table S2). This underscores the central role of coagulation dysregulation in the pathogenesis of severe disease and aligns with previous studies that have highlighted the hypercoagulable state as a hallmark of severe COVID-19, leading to thromboembolic events and multi-organ failure^1,2^. To further resolve the heatmap and provide a more detailed view of gene-specific variability, we generated boxplots of the 52 significant differentially expressed genes (Supplementary Figure S1). These visualizations confirm significant upregulation of several prothrombotic genes in severe COVID-19 while also highlighting notable inter-patient variability.

Interestingly, when correlating gene expression levels with clinical laboratory parameters, CRP but not Fibrin-D-dimer or platelet counts was significantly associated with the expression of multiple coagulation genes, including GP9, SERPINE1, ITGB3, MMRN1, and F2RL3 in the severe group (FDR < 0.05; Supplementary Table S3; Supplementary Figure S2). No significant association was noted in the mild group. This suggests that while Fibrin-D-dimer and platelet elevations likely reflect systemic activation at the protein level, CRP levels may align more closely with transcriptional regulation of platelet and endothelial pathways during inflammation.

The heterogeneity observed in the gene expression pattern, even among individuals with severe disease, highlights variability in the response and raises the possibility that additional factors, such as immune memory or autoimmunity, may modulate the response to SARS-CoV-2 infection and contribute to disease variability. Given that CRP, but not Fibrin-D-dimer or platelet counts showed correlation with a subset of coagulation-related genes, systemic inflammation may partially explain the transcriptional variation. We therefore next performed autoantibody profiling against coagulation-related antigens to investigated whether such immune signatures could underlie or complement transcriptional landscape.

### Autoantibody profiling in COVID-19 patients

**Fig. 2:**
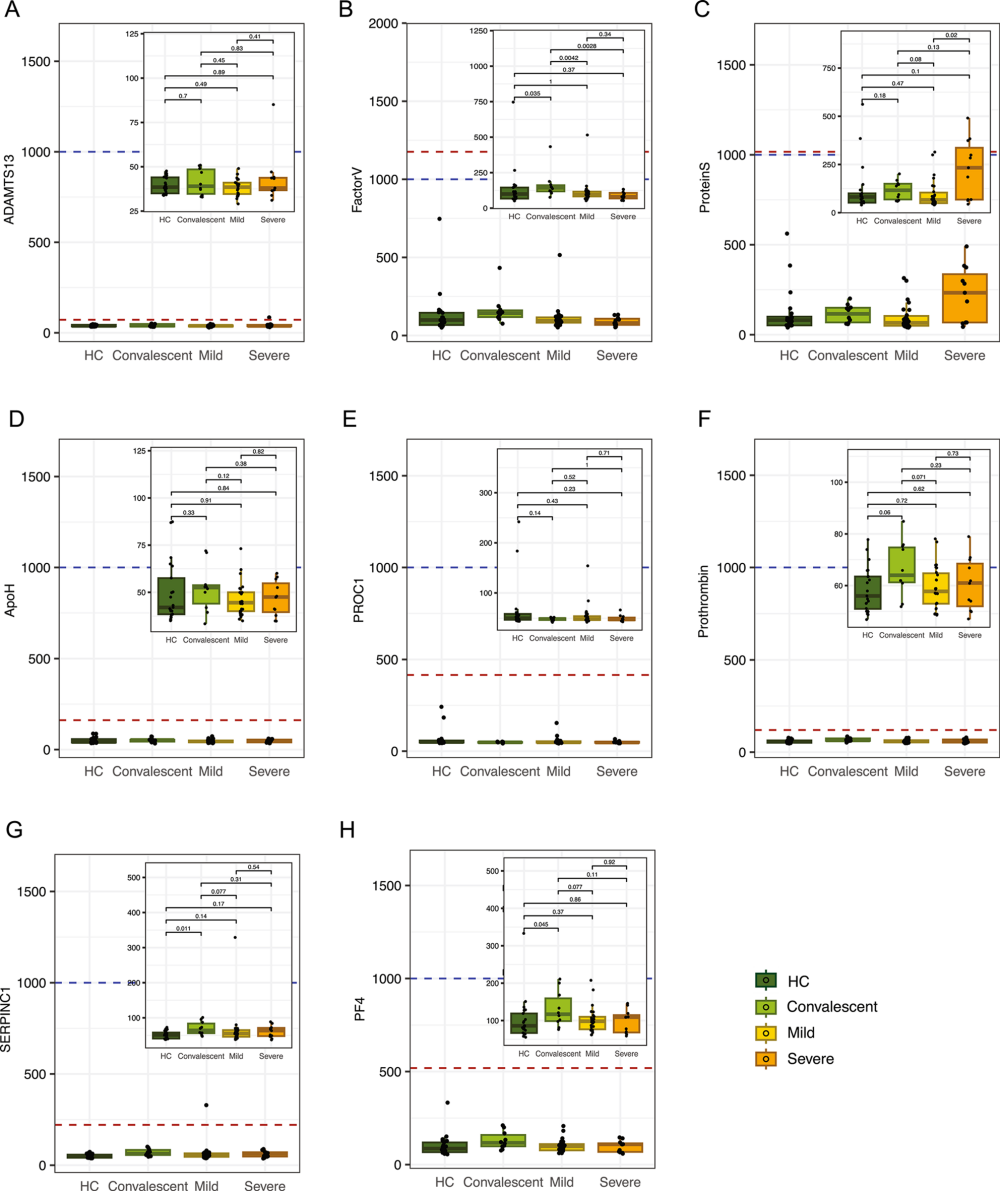
Coagulation related autoantibody profiling in COVID-19 patients. Analysis of autoantibodies against ADAMTS13 **(A)**, Factor V **(B)**, Protein S **(C)**, Apo-H **(D)**, PROC1 **(E)**, Prothrombin **(F),** SERPINC1 **(G),** and PF4 **(H)** depicted through dot plots for four groups: Healthy Controls (*n *= 21), Convalescent (*n *= 10), Mild (*n *= 26), and Severe (*n *= 11) COVID-19 patients. Positivity thresholds are marked by red dotted lines, set at either the mean fluorescence intensity (MFI) plus seven standard deviations of the healthy control group (MFI + 7SD) or an arbitrary cutoff of 1000 MFI denoted by blue dotted lines, whichever is higher. All samples are subthreshold for all antigens. Autoantibody reactivities were determined using a custom-developed multiplex bead-based immunoasssay (Luminex, Luminex corp., Ausdtin, TX, USA). Statistical significance was evaluated using Mann-Whitney U tests, with p-values indicated for each comparison. Lower-range MFI plots are included as zoomed insets inside each antigen panels to provide a more resolved view of sub-threshold distributions across clinical groups.

The observed variability in gene expression patterns led us to investigate the presence of autoantibodies against coagulation factors, as they may contribute to the differential responses observed in COVID-19 patients. We focused on a predefined panel of coagulation-related autoantibodies selected based on their hypothesized or documented involvement in COVID-19-associated thromboinflammation and vaccine-induced coagulopathies. Using EDTA-Plasma samples and an in-house Luminex bead-based autoantibody-screening assay, we assessed the presence of circulating autoantibodies to eight coagulation-related factors (ADAMTS13, Factor V, Protein S, SERPINC1, Apo-H, PROC1, prothrombin, and PF4) and their relationship to the expression of genes associated with complement-coagulation related pathways and to clinical disease severity. Figure 2 shows the mean fluorescent intensity (MFI) of each of the specific autoantibodies in different disease categories. The antigen reactivities detected for coagulation-related proteins were low compared to signals typically observed for viral proteins or established antigens in this assay format. To define positivity, we applied a dual threshold approach, defined by either as the mean MFI of the healthy control (HC) group plus seven times their standard deviation (marked by the red dotted line in Fig. 2) or an arbitrary cutoff of 1000 MFI (marked by blue dotted line in Fig. 2), whichever was higher. Our analysis did not reveal autoantibody positivity for any of the tested COVID-19 patients or controls. Therefore, we consistently refer to these reactivities as unvalidated sub-threshold antigen signals or reactivities. We acknowledge that biological interpretation of these subthreshold signals is exploratory, and they may reflect either very low-level binding or non-specific background noise. Moreover, potential limitations of the multiplex-platform such as inter-assay variability and antigen and/or antibody cross-reactivity cannot be excluded. We were limited by any further validations using more sensitive assays and orthogonal confirmatory methods to clarify if there is any biological relevance of these observations. Nonetheless, we consider this a resource-efficient and data leveraging exploratory approach, especially in settings where multiple antigens can be included at virtually no additional cost, allowing identification of putative autoantibody candidates for future mechanistic validation. Future efforts may benefit from higher-sensitivity approaches or epitope-resolved techniques to clarify the significance of low-level signals. Despite the shortcomings, in pairwise comparisons without multiple testing correction of the subthreshold MFIs of the tested antigens across groups, convalescent patients exhibited significantly higher reactivity to Factor V compared to both the active COVID-19 groups and healthy controls (HC) (Mann-Whitney U test, *p* < 0.05; Fig. 2B). Patients with severe disease exhibited higher anti-protein S reactivity compared to the patients with mild disease (Mann-Whitney U test, *p* = 0.027; Fig. 2C) despite the MFIs remained below threshold levels. Interestingly, sub-threshold reactivities to SERPINC1, PF4 and prothrombin were numerically elevated in convalescent individuals compared to healthy controls (p-values of 0.011, 0.045 and 0.06, respectively; Fig. 2G, 2H and 2F respectively). This observation, although it cannot be reliably distinguished from background noise, aligns with prior studies suggesting an enrichment of these autoantibodies following SARS-CoV-2 infection^10^.

Differences in the applied statistical tests indicate the need for further investigations into autoantibodies in these groups, and these screening assay results does not contradict previous reports suggesting that autoantibodies can exacerbate the inflammatory and coagulation responses in COVID-19, potentially leading to more severe outcomes^7,9^. These observations underscore the potential for further exploratory studies to investigate autoantibodies and prevalence post-infection, emphasizing the need for a detailed analytical approach across different patient groups.

### Correlation between autoantibody reactivity data and gene expression

**Fig. 3:**
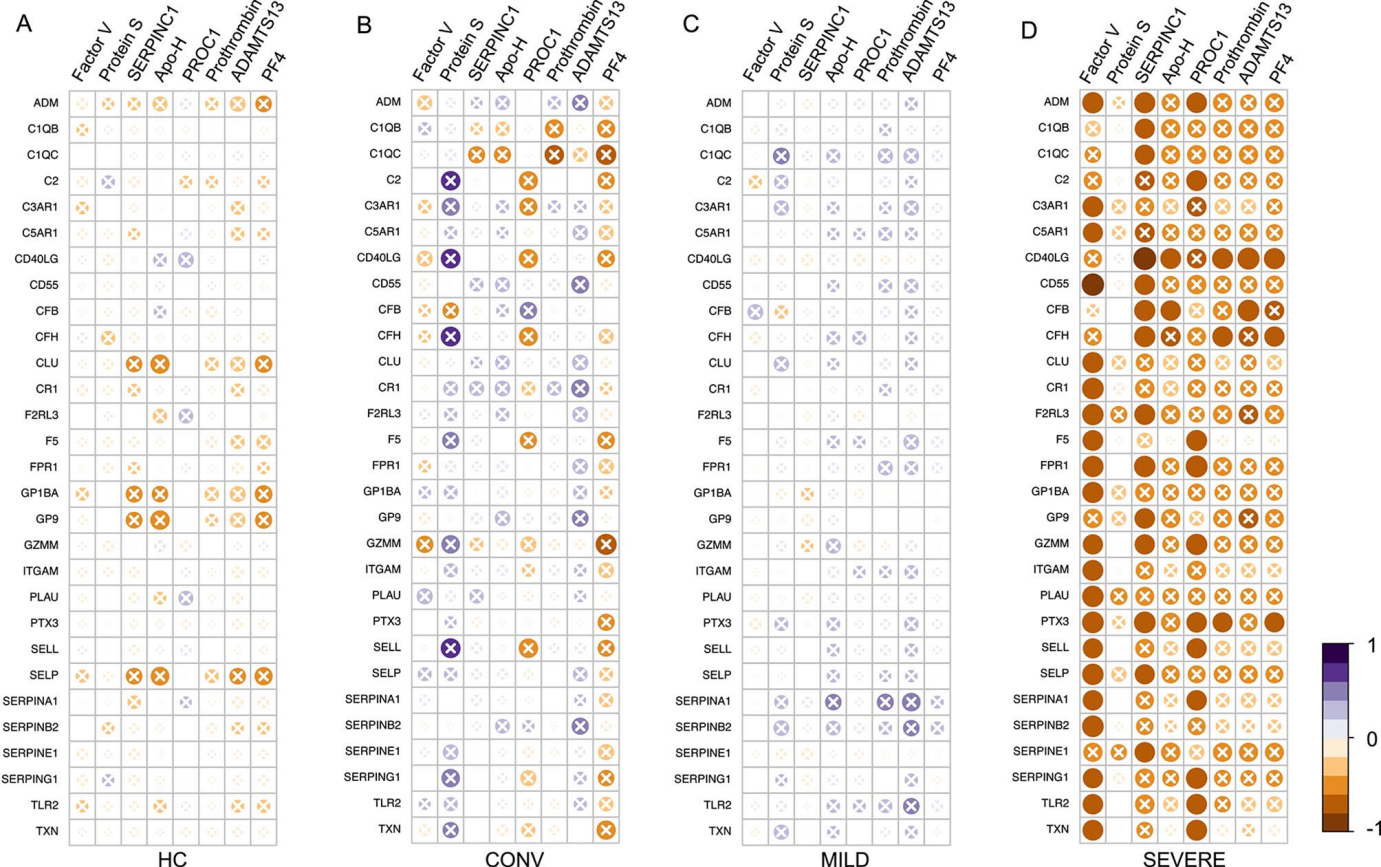
Correlation analysis between unvalidated sub-threshold autoantibody candidates and expression of significant differentially expressed Complement-coagulation-related genes. **(Panels: A-D)** Spearman correlation heatmaps showing the relationships between subthreshold autoantibody reactivities (x-axis) and expression levels of significantly differentially expressed genes (from Fig. 1) within the coagulation and complement pathways (y-axis) across different clinical groups: healthy controls (HC; *n *= 21) **(A)**, convalescent (CONV; *n *= 10) **(B)**, mild COVID-19 (MILD; *n *= 26) **(C)**, and severe COVID-19 (SEVERE; *n *= 11) **(D)**. Only genes identified as significantly dysregulated in the transcriptomic analysis (Fig. 1) were included. Each bubble represents a gene-autoantibody pair, with color intensity indicating the correlation coefficient (blue = positive, red = negative), and bubble size reflecting correlation strength. Analyses were conducted using the psych package in R, and significance was evaluated using a relaxed false discovery rate (FDR) threshold of 0.25, suitable for exploratory analysis. Non-significant correlations are marked with a white X mark inside the bubbles.

We subsequently analyzed correlations between the expression of significantly altered genes in the complement-coagulation pathways and MFIs of the autoantibody signals across different patient groups, despite no patient meeting the established positivity threshold. This exploratory approach aimed to identify subtle patterns of immune-coagulation interactions that might be biologically informative even in absence of classical autoantibody positivity. To enhance sensitivity in this exploratory context, we applied a relaxed false discovery rate (FDR) threshold of 0.25, consistent with prior exploratory immunogenomic studies that prioritize hypothesis generation over strict statistical significance^16–18^. For all correlation analyses involving subthreshold autoantibody reactivities, including those with gene expression, we adjusted p-values using an FDR approach based on the number of unique antigen targets tested in this study, rather than the full 96-analyte bead panel, which contains technical replicates and controls not representing distinct antigens^19^ (see Methods). This allowed us to identify directional trends and nominate biologically plausible candidate associations for future validation. As shown in Fig. 3, only the severe COVID-19 group demonstrated gene-autoantibody correlations that passed the adjusted FDR threshold (< 0.25), while the healthy controls (HC), convalescent and mild groups showed no such significance and visually denoted by cross-marked bubbles in the figure (Supplementary Table S4). The lack of correlation is consistent with the expected stability and regulatory effectiveness of their immune systems. In contrast, the severe COVID-19 group demonstrated a distinct pattern of widespread negative correlations between several coagulation-associated subthreshold autoantibody candidates and key regulatory genes. The observed significance-limited to the severe group (adj. p-value < 0.25; Fig. 3D) underscores the importance of context-specific immune dysregulation in driving these interactions. These correlations involved both immune-modulatory genes and endothelial or platelet-associated markers, and while modest in strength (R typically between − 0.65 and − 0.85), they consistently met the adjusted FDR threshold. Genes such as CD40LG, PTX3, FPR1, ADM, TXN, SERPING1, SERPINE1, and CFB showed reproducible associations with subthreshold reactivates targeting ADAMTS13, Factor V, SERPINC1, PROC1, Prothrombin, ApoH, and PF4, all adjusted p-value < 0.25 (Supplementary Table S4). For instance, CD40LG expression negatively correlated with five distinct candidate antigens, including ADAMTS13 (*R* = − 0.79, adj. p-value = 0.236), SERPINC1 (*R* = − 0.85, adj. p-value = 0.236), Prothrombin, Apo-H, and PF4, suggesting it may be a convergence point in immune-coagulative dysregulation in severe disease. Similarly, PTX3, a marker of vascular inflammation and predictive marker of COVID-19 mortality^20^, was negatively associated with both Factor V and PROC1 subthreshold reactivities, while FPR1, involved in neutrophil chemotaxis and linked to pulmonary pathology in severe COVID-19^21^, exhibited strong inverse correlations with Factor V, SERPINC1, and PROC1, all within the exploratory significance threshold. These patterns reinforce a broader observation that severe disease may potentially be characterized by immunological interference with transcriptional regulation of vascular and hemostatic genes, even when classical thresholds for autoantibody positivity are not reached. Importantly, no similar correlations were observed in the other clinical groups, supporting the idea that these transcription-autoantibody reactivity associations might be more than merely reflective of background immune activation, but rather reflect a disease-specific breakdown in regulatory control during severe SARS-CoV-2 infection.

Interestingly, although Protein S reactivity levels (below threshold) were higher in severe cases than in mild cases (Fig. 2C), correlations with the transcriptional profile were absent (Fig. 3D). This lack of correlation suggests that the pathogenic role of these autoantibodies, if any, may be independent of direct transcriptional changes in coagulation-related genes, potentially acting through post-translational modifications or interactions with other immune pathways that were not captured in this analysis.

To test whether these candidate gene-autoantibody associations reflected general markers of systemic inflammation or coagulation activity, we next examined correlations between subthreshold autoantibody MFI values and routine clinical laboratory parameters CRP, Fibrin-D-dimer, and platelet counts in both mild and severe COVID-19 cases. Notably, no significant associations were observed with any of these markers, even using a relaxed FDR threshold of 0.25 (Supplementary Table S5). This potentially suggests that the observed transcriptional correlations are not merely surrogate reflections of systemic inflammatory load or coagulative imbalance. Instead, they may represent a more specific immunotranscriptional interaction unique to the severe disease state.

The disparate correlation patterns observed between mild and severe COVID-19 cases leads us to speculate unique underlying pathogenic mechanisms tailored to the intensity of the disease. In severe cases, the predominantly negative correlations might reflect a tipping point where homeostatic mechanisms are overwhelmed, leading to uncontrolled coagulation and systemic inflammation. This scenario indicates a possible over-activation of immune responses that could drive the severity of clinical manifestations. Conversely, the milder cases display a pattern of more favorable or neutral correlations, hinting at a more regulated and balanced interplay between the immune and coagulation systems. This controlled response potentially curtails the escalation of the disease, helping to maintain physiological stability and prevent the progression to severe forms of the illness. The lack of adjusted p-values < 0.05, despite noticeable R values, suggests that while trends in the data exist, they do not reach classically accepted statistical significance. This highlights the need for cautious interpretation of these correlations and further investigation into their biological impact on disease progression in COVID-19.

To summarize these findings, severe COVID-19 patients uniquely exhibited reproducible, FDR-filtered correlations between coagulation gene expression and subthreshold autoantibody reactivities, tempting us to speculate that even subthreshold autoantibody reactivities may interact with the host transcriptome in a context-dependent manner. While these associations remain exploratory, they highlight the potential for autoantibody-transcriptional crosstalk as a contributing factor to thromboinflammation in severe disease. This underscores the importance of further mechanistic studies especially integrating transcription, protein regulation, and translational dynamics to unravel the immune-coagulation interface in COVID-19 and similar inflammatory syndromes.

### Multi-omics correlations across antigens, transcriptomics, proteomics, and clinical laboratory parameters

Building on the observed associations between subthreshold autoantibody reactivities and transcriptional alterations in severe COVID-19, we next evaluated whether these relationships extended to the plasma proteome and clinically relevant inflammatory markers. Given that several genes associated with autoantibody profiles encode secreted immune or vascular proteins, we hypothesized that these transcriptional and humoral signatures may also manifest in systemic proteomic changes. To investigate this, we performed correlation analyses integrating previously generated and published Olink plasma proteomics data^22^ with both subthreshold autoantibody MFI and RNA-seq expression profiles across patient subgroups. This approach aimed to uncover potential links between serologic dysregulation, gene expression, and immune-coagulatory markers at the protein level. In addition, we assessed whether these molecular interactions aligned with conventional clinical indicators of inflammation and thrombosis.

**Fig. 4:**
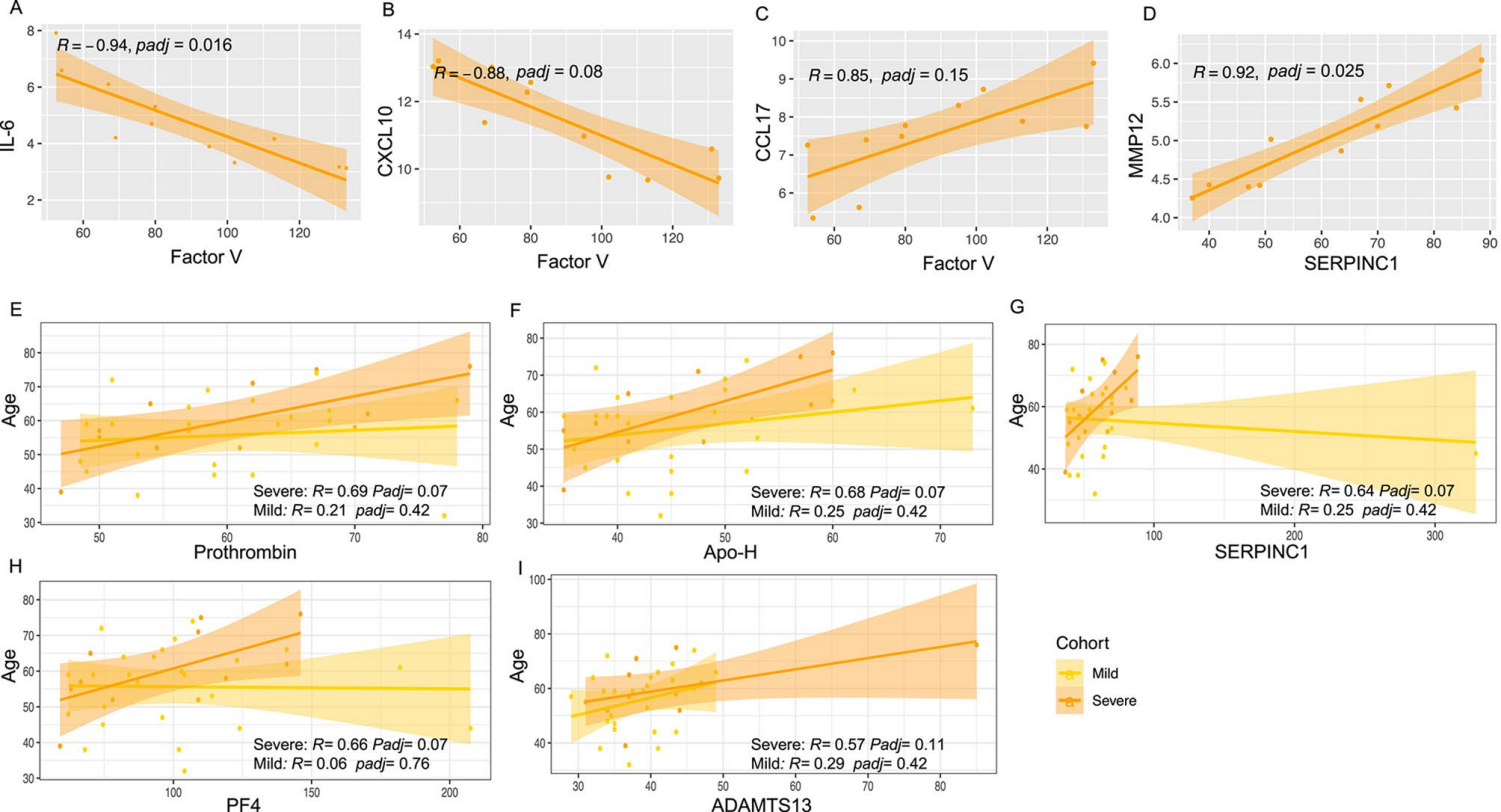
Correlation between antigen reactivity, age, and circulating proteins in COVID-19. **(Panels: A-D)** Representative scatter plots highlighting significant correlations between unvalidated subthreshold autoantibody candidates (MFI; x-axis) and Olink-measured plasma protein abundance (NPX values; y-axis) in the COVID-19 severe group. In the represented graphs, Factor V reactivity is showing negative correlation with plasma protein IL-6 **(A)** and CXCL10 **(B)**, and positive correlation with CCL17 **(C)**, and for SERPINC1 reactivity showing positive correlation with plasma protein MMP-12 **(D)**. Each dot represents one individual across the COVID-19 cohort. Trend lines and shaded confidence intervals are shown. All correlation coefficients (R) and adjusted p-values (padj) are indicated. An exploratory false discovery rate (FDR) threshold of 0.25 was applied to define significance and only features with FDR-adjusted *p* < 0.25 are presented. **(Panels: E–I)** Scatter plots showing Spearman correlations between age and subthreshold MFI levels of IgG autoantibodies against Prothrombin **(E)**, Apo-H **(F)**, SERPINC1 **(G)**, PF4 **(H),** and ADAMTS13 **(I)** in COVID-19 patients. Each dot represents an individual; best-fit trend lines with 95% confidence intervals are overlaid. Data are color-coded by disease severity (Mild: yellow; Severe: orange). Correlation coefficients (R), p-values, and Benjamini-Hochberg adjusted p-values (padj) are indicated.

Focusing first on autoantibody-protein correlations, we analyzed relationships between subthreshold median fluorescence intensity (MFI) values of coagulation-related antigens and levels of 92 plasma proteins measured using the Olink Immuno-Oncology panel. Applying an exploratory FDR threshold of 0.25 appropriate for hypothesis-generating analyses, we identified several significant correlations in the severe disease group (Fig. 4A–D; Supplementary Table S6). For instance, subthreshold anti-Factor V antigen reactivates correlated negatively with IL-6 (*R* = − 0.94, adj. p-value = 0.016) and CXCL10 (*R* = − 0.88, adj. p-value = 0.081) (Fig. 4A and B), cytokines previously implicated in cytokine storm and adverse COVID-19 outcomes^23^. A positive correlation was also observed between subthreshold anti-Factor V and CCL17 (*R* = 0.85, adj. p-value = 0.15) (Fig. 4C), a chemokine reported to be suppressed in severe COVID-19 and proposed as an early triage marker for pneumonia risk^24^. While the functional impact of these associations remains to be clarified, these findings suggest that even low-level autoantibody reactivities may reflect or contribute to broader inflammatory chemokine modulation in severe disease. Similarly, subthreshold anti-SERPINC1 autoantibodies correlated positively with MMP-12 (*R* = 0.92, adj. p-value = 0.025) (Fig. 4D), a macrophage-derived metalloproteinase linked to tissue remodeling and lung injury^25^, indicating potential crosstalk between humoral immunity and extracellular matrix degradation pathways. These associations were not observed in the mild group and were sparse in convalescent individuals, although in the latter, a strong correlation was noted between subthreshold anti-ADAMTS13 and MCP-2 (*R* = 0.95, adj. p-value = 0.022) as shown in Supplementary Figure S3, possibly reflecting late-phase myeloid or endothelial remodeling.

We next assessed correlations between RNA-seq gene expression and Olink proteomic markers applying a stringent significance threshold of adjusted p-value < 0.05 to prioritize robust and mechanistically interpretable associations, focusing on genes previously identified as differentially expressed and enriched for complement and coagulation-related functions. Unexpectedly, the mild disease group exhibited a greater number of significant gene-protein correlations than the severe group (adj. p-value < 0.05; Supplementary Table S7) suggesting a more coordinated regulation of transcription and systemic protein expression in less advanced disease. As shown in Supplementary Figure S4A, several genes, including SERPING1, TXN, F5, ITGA2B, and C3AR1, demonstrated robust positive correlations with Olink proteins involved in immune signaling, vascular homeostasis, and leukocyte recruitment. For example, SERPING1 expression strongly correlated with IFN-γ (*R* = 0.90, adj. p-value < 0.0001), IL-10 (*R* = 0.78, adj. p-value = 0.0019), and CXCL10 (*R* = 0.68, adj. p-value = 0.015), implicating its potential involvement in modulating interferon-stimulated and regulatory cytokine axes. Similarly, TXN expression was associated with VEGFA, CCL23, IL-6, and CASP-8, highlighting its connection to redox regulation and inflammatory vascular remodeling. Genes such as C3AR1 and TLR2 also displayed coordinated expression with VEGFA, CSF-1, and IL-10, reinforcing the interplay between complement signaling, myeloid activation, and cytokine regulation. In contrast, gene-protein correlations in severe disease were fewer and less robust with only plasma TNFRSF4 showing negative correlations with C1RL, SELL, SERPINA1, and TLR2 genes (adj. p-value < 0.05; *R* <– 0.9) (Supplementary Figure S4B), possibly reflecting disrupted transcriptional-proteomic coupling due to immune exhaustion, compartmentalization, or systemic dysregulation in critical illness.

Finally, to relate these molecular layers to clinical inflammation and coagulation markers, we examined correlations between Olink proteins and clinical laboratory parameters including C-reactive protein (CRP), Fibrin-D-dimer, and platelet count (Supplementary Table S8). While most associations did not reach significance, two notable and severity-specific relationships emerged. In the mild group, CRP negatively correlated with TRAIL (*R* = − 0.69, adj. p-value < 0.05) (Supplementary Figure S5A), a TNF superfamily member involved in apoptosis and antiviral responses. Prior studies have mostly linked TRAIL to protective immune regulation during viral infections^26^, and its inverse relationship with CRP here suggests a higher TRAIL levels may contribute to attenuated inflammation in mild disease contexts. In severe disease, a strong positive correlation was observed between Fibrin-D-dimer and MUC16 (*R* = 0.96, adj. p-value < 0.05) (Supplementary Figure S5B). MUC16 (CA125) is a mucin-like glycoprotein primarily known for its role in epithelial integrity and has been implicated in immune modulation in cancer and inflammatory contexts^27^. Notably, while prior studies have reported higher MUC16 mRNA expression in mild compared to severe COVID-19^28^, our data show a strong positive correlation between plasma MUC16 protein levels and D-dimer specifically in severe cases. This apparent discrepancy may reflect post-transcriptional regulation, increased epithelial shedding, or systemic release of MUC16 in response to endothelial or mucosal barrier disruption during hyperinflammatory and hypercoagulable states.

Together, these multi-omic correlations across transcriptomics, autoantibody profiling, proteomics, and clinical markers support the hypothesis that subthreshold humoral immune responses, despite remaining below conventional diagnostic cutoffs, may nonetheless associate with coherent immuno-coagulatory signatures in severe disease. In mild cases, more synchronized transcript-protein interactions may reflect preserved immune homeostasis, while severe disease appears characterized by fragmented but selective cross-layer associations involving endothelial stress, inflammation, and coagulopathy. These findings highlight the utility of integrative, quantitative approaches in uncovering latent dimensions of immune dysregulation in COVID-19, and underscore the importance of context and disease phase and severity in interpreting autoantibody and proteomic profiles.

### Age-dependent autoantibody reactivity

Our study further explored the relationship between age and autoantibody reactivities across different severities of COVID-19 at subthreshold MFI signals, focusing on potential trends (using an exploratory FDR < 0.25) rather than diagnostic positivity. In all the subthreshold autoantibodies tested in our panel we observed strong positive correlations between age and the subthreshold MFI of candidate reactivities against Prothrombin (*R* = 0.68, *p* = 0.02, adj. p-value = 0.07), Apo-H (*R* = 0.68, *p* = 0.021, adj. p-value = 0.07), SERPINC1 (*R* = 0.64, *p* = 0.035, adj. p-value = 0.07), PF4 (*R* = 0.66, *p* = 0.03, adj. p-value = 0.07),and ADAMTS13 (*R* = 0.57, *p* = 0.07, adj. p-value = 0.11) in severe cases (Fig. 4E–I). These correlations were consistently stronger in severe patients than in mild cases, where age-dependent reactivities to the same antigens were weaker and statistically non-significant (adj. p-value > 0.42, *R* < 0.3). Interestingly, when analyzing all COVID-19 patients together, despite lower correlation coefficients, certain subthreshold autoantibody candidates such as Prothrombin, Apo-H, ADAMTS13, and SERPINC1 approached conventional significance thresholds, (adj. p-value < 0.06, R *≥* 0.37) (Supplementary Table S9). This indicates that while individual correlations of subthreshold reactivities may appear modest, collectively they may reveal significant age-related trends across the COVID-19 spectrum.

Although these findings emphasize the complex influence of age on immune system behavior, suggesting that older patients might have distinct immune profiles that could impact their response to COVID-19, it is important to emphasize that these MFI values remained below established positivity thresholds, and while adjusted p-values suggest statistical associations, the biological implications remain exploratory. These results should therefore be interpreted as hypothesis-generating and not diagnostic. This observation aligns with previous studies suggesting that aging is associated with an increase in autoantibody production due to immunosenescence and inflammaging, contributing to the exacerbated clinical manifestations of diseases like COVID-19^8^. For example, older COVID-19 patients frequently exhibit a more severe disease course, characterized by enhanced inflammatory responses and a propensity toward autoreactivity, which may enhance hypercoagulability and increase the risk of thromboembolic events^9^. The correlation of these autoantibodies with disease severity underscores the need for age-stratified analysis and potentially also future therapeutic strategies that address both immune dysregulation and the heightened coagulation state observed in the elderly^29^. The higher prevalence of autoantibodies in older patients^30^ accentuates the need to integrate immune modulation and anticoagulation therapies into COVID-19 management strategies specifically designed for this demographic. While our findings do not suggest clinical utility of these low-reactive subthreshold autoantibody candidates in their current form, they point to age-related immune patterns worth investigating further. Future research should delve deeper into how aging affects autoantibody production including new-onset ones that emerges following infection^31^ and its implications for disease progression and therapeutic outcomes in elderly COVID-19 patients.

Our research contributes to the growing body of knowledge on the critical role of autoantibodies in the pathophysiology of COVID-19, especially in severe cases. We observed that while several subthreshold antigen reactivities were negatively correlated with coagulation-related gene expression, they also exhibited both negative (IL-6 and CXCL10) and positive (MMP-12 and CCL17) correlations with plasma proteins linked to inflammation, endothelial stress, and matrix remodeling. While these associations may suggest potential immuno-coagulatory perturbations, the detected reactivities were below established positivity thresholds and remain unvalidated. These inverse associations may also reflect non-causal relationships, such as immune exhaustion or broader epiphenomena associated with severe disease, rather than indicating a direct immunomodulatory role. Therefore, we interpret these findings as exploratory and hypothesis-generating, warranting further mechanistic investigation in targeted follow-up studies. This finding highlights the complexity of immune interactions in COVID-19 and supports the need for further studies to elucidate the mechanisms by which autoantibodies affect disease progression and outcomes, especially in long-term sequelae showing persistent complement dysregulation and thromboinflammation, as recently noted by Cervia-Hasler et al. (2024)^32^. Understanding these interactions could lead to the development of targeted therapies and exploratory biomarker candidates for disease severity, enhancing our management strategies for both acute and prolonged manifestations of COVID-19. In conclusion, this study underscores the potential of incorporating both autoantibody profiling and gene expression analysis in conjunction with plasma proteomics to dissect the complex pathogenesis of severe COVID-19. While the direct impact of autoantibodies on disease processes requires further validation, our findings hint at a potential relationship between subthreshold antigen reactivities and disease severity, suggesting that they might influence a range of intriguing pathophysiological pathways. The presence of coherent multi-layered correlations despite subclinical autoantibody reactivities supports the hypothesis that these responses may participate in shaping immune and vascular dynamics in a context-dependent manner. These insights may inform future studies and therapeutic strategies aimed at modulating immune responses to improve clinical outcomes in severe COVID-19 cases. Further research will be crucial to validate and establish the clinical relevance of any candidate autoantibodies and their utility in guiding interventions for COVID-19, particularly in severe and long-term scenarios.

## Methodology

### Study cohort

 The cohort of COVID-19 patients and healthy controls used in this study has been previously described in detail^15,22^. Briefly, the study included 37 hospitalized COVID-19 patients who tested positive for SARS-CoV-2, stratified by oxygen consumption into two groups: mild (O2 consumption < 4 l/min; *n* = 26) and severe (O2 consumption ≥ 4 l/min; *n* = 11). Exclusion criteria included significant pre-existing conditions (liver cirrhosis, severe renal insufficiency, chronic obstructive pulmonary disease, and chronic lung diseases leading to habitual SpO2 ≤ 92%). Additionally, 31 healthy controls (HC) were included, with 10 testing positive for SARS-CoV-2 antibodies, referred to as Convalescent (Conv). All study participants provided informed consent, with procedures approved by the regional ethics committees of Stockholm (dnr 2020 − 01865) and conducted in accordance with the Declaration of Helsinki. Clinical laboratory parameters including CRP, Fibrin-D-dimer, and platelet counts were extracted retrospectively from hospital records in a blinded manner.

### Autoantibody screening

 The panel of coagulation-related proteins selected for autoantibody profiling, ADAMTS13, Factor V, Protein S, SERPINC1 (antithrombin III), Apo-H (β2-glycoprotein I), PROC1 (Protein C), prothrombin, and platelet factor 4 (PF4) was originally selected for a parallel study investigating autoimmune responses following COVID-19 vaccination^33^. These targets were chosen based on their known roles in thrombotic diseases such as thrombotic thrombocytopenic purpura (TTP) and antiphospholipid syndrome, as well as their established functions as key regulators of coagulation. For example, ADAMTS13 autoantibodies are characteristic of acquired TTP, while Apo-H and prothrombin are commonly targeted in antiphospholipid syndrome. Deficiencies in Protein S, Protein C, and SERPINC1 are linked to inherited thrombophilia. The inclusion of PF4 was based on its clinical relevance in vaccine-induced immune thrombotic thrombocytopenia (VITT)^33^. The methodology for detecting human IgG autoantibodies, previously described^19^, includes the use of magnetic beads (MagPlex^®^, Luminex Corp.) prepared using the AnteoTech Activation Kit for Multiplex Microspheres to ensure effective coupling with specific antigenic proteins. The beads were coupled with commercial proteins, including ADAMTS13, Factor V, Protein S, SERPINC1, Apo-H, PROC1, Prothrombin, and PF4, at standardized coupling ratios (1.5 × 10^6^ beads per 3 µg of protein). Stored plasma samples obtained from the patients were initially diluted in PBS (1:25), followed by a secondary dilution (1:10) in PBS with 0.05% Tween, 3% BSA, and 5% Milk. The bead-sample mixture was then incubated at 650 rpm for two hours. Following three wash cycles in PBS with 0.05% Tween, the beads were fixed in 0.2% PFA for 10 min. Detection was carried out using a secondary antibody from Invitrogen, with incubation lasting 30 min before analysis on a FlexMap 3D^®^ instrument (Luminex Corp, Austin, TX, USA).

### Transcriptomics data analysis

 Whole blood transcriptomics data for the analysis was sourced from the previously published study by the group^15^. Transcriptomics data was available for all healthy controls (*n* = 31 including 10 convalescent), and for mild (*n* = 26) and severe (*n* = 11) COVID-19 cases included in this study. The data were reanalyzed, focusing on differential gene expression in a curated set of genes related to the coagulation and complement cascades. Two-hundred and thirty-three genes were compiled by integrating genes annotated in established pathway databases, including KEGG (hsa04610: Complement and Coagulation Cascades) and PANTHER (P00011: Blood Coagulation - Homo sapiens) retrieved via Enrichr libraries^34^ (https://maayanlab.cloud/Enrichr/), WikiPathways (WP2806, WP545, WP558) were directly retrieved from the WikiPathways database (https://www.wikipathways.org), Gene Ontology Biological Process (GO:0007596 - Blood Coagulation, GO:0006956 - Complement Activation), and Reactome (R-HSA-166658 - Complement Cascade, R-HSA-977606 - Regulation of Complement Cascade) were retrieved from MSigDB (v2023.1) using the GSEA/MSigDB interface (https://www.gsea-msigdb.org).

Differential gene expression analysis was conducted using the R package DESeq2 v1.44.0^35^. To adjust for potential biases, characteristics such as age, gender, BMI, and other unwanted variations, as identified by the RUVSeq package v1.38.0^36^, were included in the model matrix. Genes with an adjusted p-value (adj. p) less than 0.05 and a log2 fold change greater than one was considered significantly regulated. Principal component analysis (PCA) was performed with the PCA tools package v2.16.0 in R, using transcripts per million (TPM) normalized data from the complement and coagulation cascade genes. The Wilcoxon test was executed using the stat_compare_means function in R. Spearman’s correlation was calculated using the corr.test function from the psych package v2.4.3. Genes with a variance across samples below 0.1 were excluded from the correlation analysis. Corrections for multiple hypothesis testing were applied using the Benjamini-Hochberg method following the correlation analysis. Visualization of PCA dimensions and the generation of dot plots were carried out using the ggplot2 package v3.5.1 in R. The heatmap was generated using the ComplexHeatmap package v2.20.0, and the corrplot was created with the corrplot package v0.92.

### Plasma proteomics data extraction (Olink platform)

 Plasma proteomic profiling for this cohort was previously conducted using the Olink^®^ Immuno-Oncology Target 96 panel, as reported in detail in the original study^22^. The platform employs proximity extension assay (PEA) technology to measure relative expression levels of 92 inflammation- and immunity-related proteins in plasma. Data were reported as Normalized Protein eXpression (NPX) values, which are log₂-scaled, normalized units reflecting relative protein abundance, adjusted for inter- and intra-plate variability using Olink’s internal controls. In the present study, pre-processed NPX values were extracted specifically for downstream correlation analyses with transcriptomic, autoantibody profiling, and clinical laboratory data. No additional normalization or transformation was applied. Only proteins passing quality control in the original dataset were included in the analysis.

### Multi-omics correlation analysis

 To evaluate cross-layer associations, multi-omic correlation analyses were performed linking candidate autoantibody MFI values (measured via custom multiplex bead-based immunoasssay), Plasma protein levels (Olink Immuno-Oncology Target 96 platform), whole blood transcript levels (RNA-seq) and clinical laboratory parameters (CRP, Fibrin-D-dimer, platelets). For statistical correction of multiple comparisons, we considered only the number of unique antigens targets tested in our panel used in this study rather than the total number of 96 analytes included in the Luminex-based assay, which included a combination of antigen-coupled beads, technical replicates, and internal assay controls (e.g., uncoupled beads, anti-human IgG) as described in our previous study^19^. Several bead IDs were linked to the same antigen for consistency assessment, and some were included as non-antigen controls. This antigen-specific correction strategy reflects the biological intent of the comparisons.

Autoantibody-gene correlations focused on a curated list of genes associated with coagulation and complement pathways, compiled from multiple sources, including the KEGG coagulation and complement cascade gene sets (KEGG:04610), Reactome, and Gene Ontology (GO:0007596, GO:0006956), merged to ensure pathway relevance. Only genes with sufficient expression variance (variance > 0.1 across samples) were retained for analysis. For exploratory autoantibody analyses, a false discovery rate (FDR) cutoff of 0.25 was applied due to the subthreshold nature of autoantibody signals and the hypothesis-generating objective.

Autoantibody-Olink correlations were calculated using Spearman’s correlation between MFI values and normalized protein expression (NPX) values. As all autoantibody signals remained below positivity thresholds, an exploratory FDR cutoff of 0.25 was used to identify hypothesis-generating associations.

Gene-Olink correlations were computed separately for each patient group (mild, severe, convalescent), using Spearman’s correlation between log₂-transformed TPM counts and NPX values. For this analysis, a conservative adjusted p-value < 0.05 was applied to prioritize robust associations.

Olink-clinical laboratory parameter correlations were performed using the same statistical framework. Associations with adjusted p-value < 0.05 were considered significant.

All statistical tests were corrected using the Benjamini-Hochberg method. Correlation heatmaps and chord plots were generated using ComplexHeatmap, corrplot, and circlize packages in R.

### Data limitations and perspectives

This study provides an integrative view of complement-coagulation related gene expression and antigen reactivities across the COVID-19 disease spectrum. However, the subthreshhold reactivities of the autoantibody candidates and the absence of validated autoantibody positivity limits the ability to establish causative and mechanistic links. Our findings highlight exploratory subthreshold reactivities and gene expression changes that may be associated with disease severity, but these observations warrant further validation in larger independent cohorts. Moreover, the current platform’s sensitivity may be insufficient to detect low-affinity or low-abundance autoantibodies; thus, future studies should apply more sensitive and orthogonal validation methodologies, to clarify the biological relevance of these subthreshold signals. The inclusion of plasma proteomic markers and clinical laboratory parameters provides complementary evidence supporting immune-coagulation dysregulation, though the cross-sectional nature of the data and limited sample size constrain longitudinal interpretation. Future research should explore post-translational modifications and include longitudinal sampling and functional assays to better elucidate the complex immune-coagulation dynamics and its contribution to COVID-19 pathophysiology.

## Supplementary Information

Below is the link to the electronic supplementary material.

Supplementary material 1 (PDF 913.2 kb)

Supplementary material 2 (XLSX 16.4 kb)

Supplementary material 3 (XLSX 15.7 kb)

Supplementary material 4 (XLSX 24.5 kb)

Supplementary material 5 (XLSX 85.3 kb)

Supplementary material 6 (XLSX 12.7 kb)

Supplementary material 7 (XLSX 137.3 kb)

Supplementary material 8 (XLSX 599.9 kb)

Supplementary material 9 (XLSX 32.6 kb)

Supplementary material 10 (XLSX 10.6 kb)

## Data Availability

The datasets generated and analyzed during the current study are available from the corresponding author on reasonable request.
