# Supplementary material for: Immune-coagulation dynamics in severe COVID-19 revealed by autoantibody profiling and multi-omics integration
Source: Sci Rep. 2025 Sep 1;15:32149. doi: 10.1038/s41598-025-17054-6 (PMC12402181; doi:10.1038/s41598-025-17054-6)
Supplement: Supplementary file 1 — Supplementary material 1 (PDF 913.2 kb) [file 41598_2025_17054_MOESM1_ESM.pdf]

Supplementary Figures:

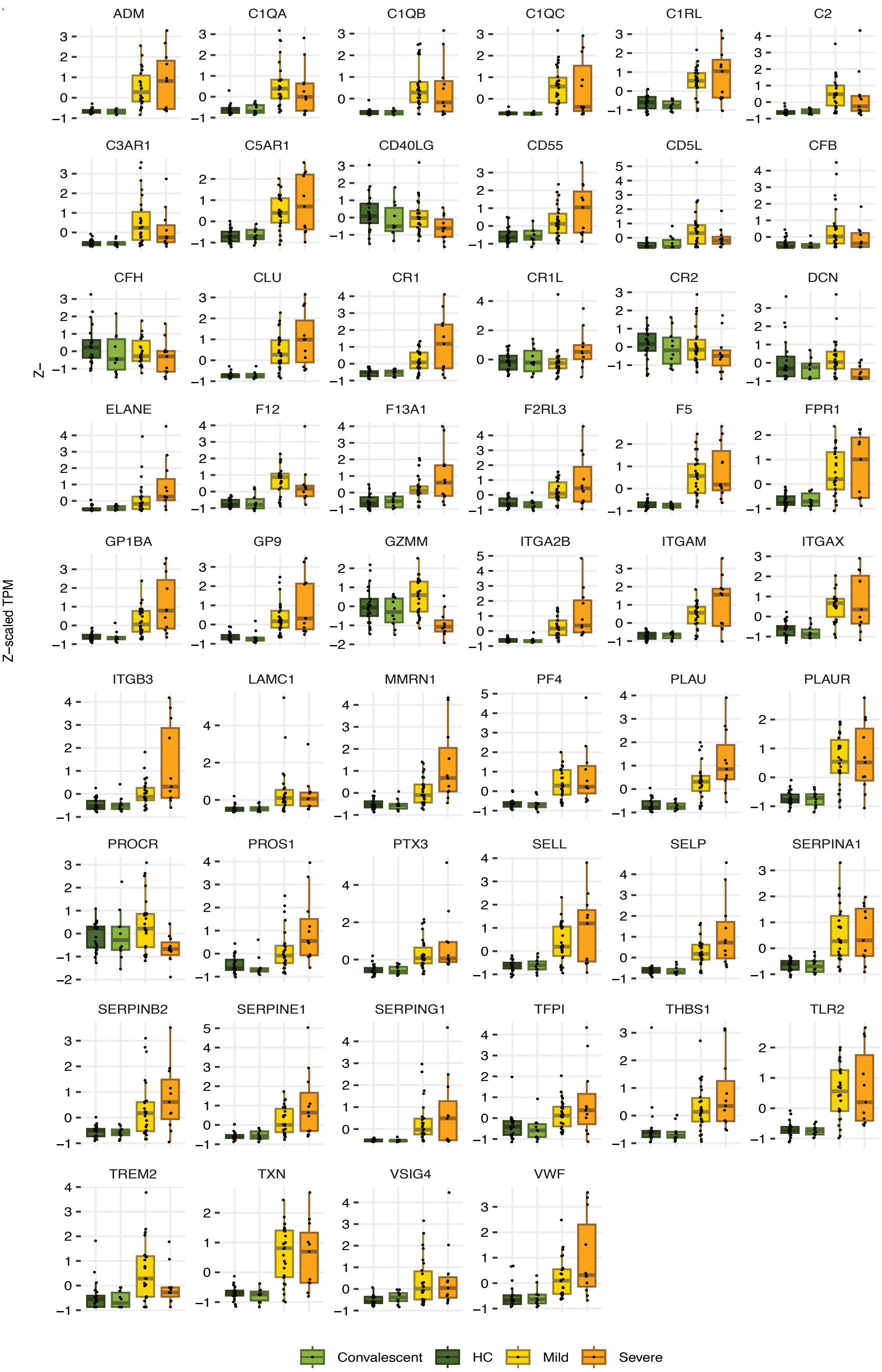

Supplementary Figure S1: Distribution of complement-coagulation gene expression across patient groups. Boxplots display z-score scaled transcript per million (TPM) expression values for 52 differentially expressed genes associated with complement and coagulation pathways across healthy controls (HC), convalescent individuals, and patients with mild or severe COVID-19. Each plot illustrates group-wise expression variability for individual genes, offering a detailed overview of transcriptional perturbations irrespective of statistical significance.

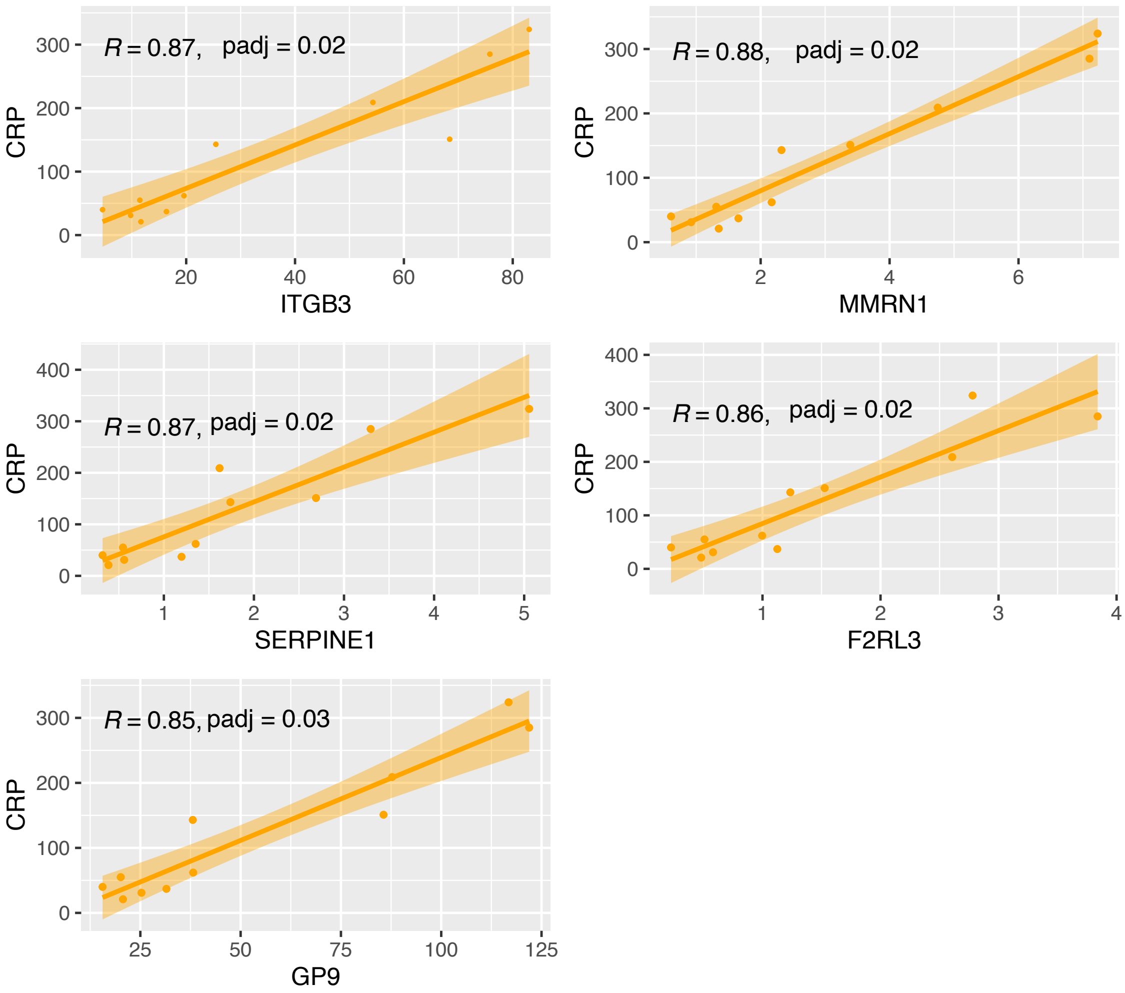

Supplementary Figure S2: Correlation between complement-coagulation gene expression and CRP in severe COVID-19. Scatter plots showing significant positive correlations between CRP (C-reactive protein) levels (mg/L; y-axis) and transcript expression (TPM; x-axis) of selected complement-coagulation pathway genes (ITGB3, MMRN1, SERPINE1, F2RL3, GP9) in patients with severe COVID-19. Each plot includes the Spearman correlation coefficient (R) and FDR-adjusted p-value (padj). An adjusted p-value of <0.05 was considered statistically significant. These associations suggest a potential link between inflammatory burden and coagulation gene activation during severe disease.

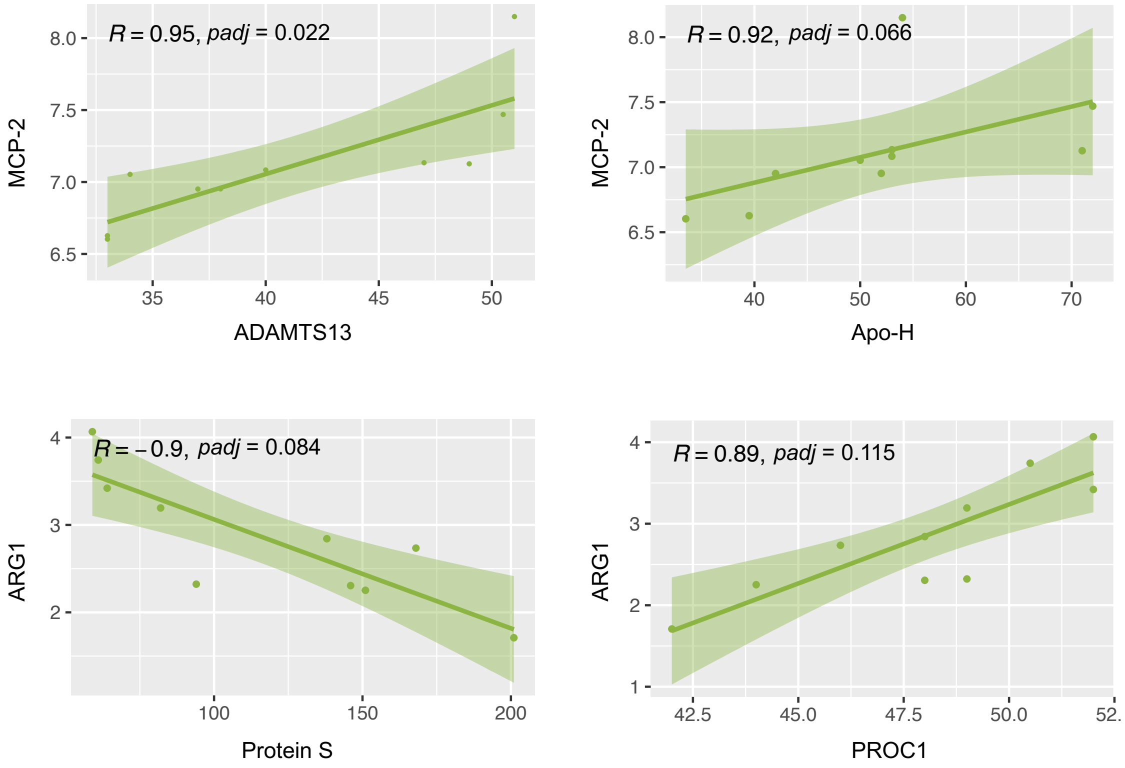

Supplementary Figure S3. Selected correlations between antigen reactivities and plasma protein levels in convalescent COVID-19 individuals. Scatter plots depict representative exploratory correlations between sub-threshold autoantibody candidate median fluorescence intensities (MFIs; x-axis) and plasma protein levels by Olink panel (NPX; y-axis) in convalescent patients. Notable associations include a strong positive correlation trend between anti-ADAMTS13 and MCP-2, and anti-Apo-H and MCP-2. Conversely, negative correlation trends were observed between anti-Protein S and ARG1, and anti-PROC1 and ARG1. All correlation coefficients (R) and adjusted p-values (padj) are indicated. An exploratory false discovery rate (FDR) threshold of 0.25 was applied to define significance. These findings suggest potential immune-proteomic interactions even in the absence of classical autoantibody positivity during the post-acute phase of infection.

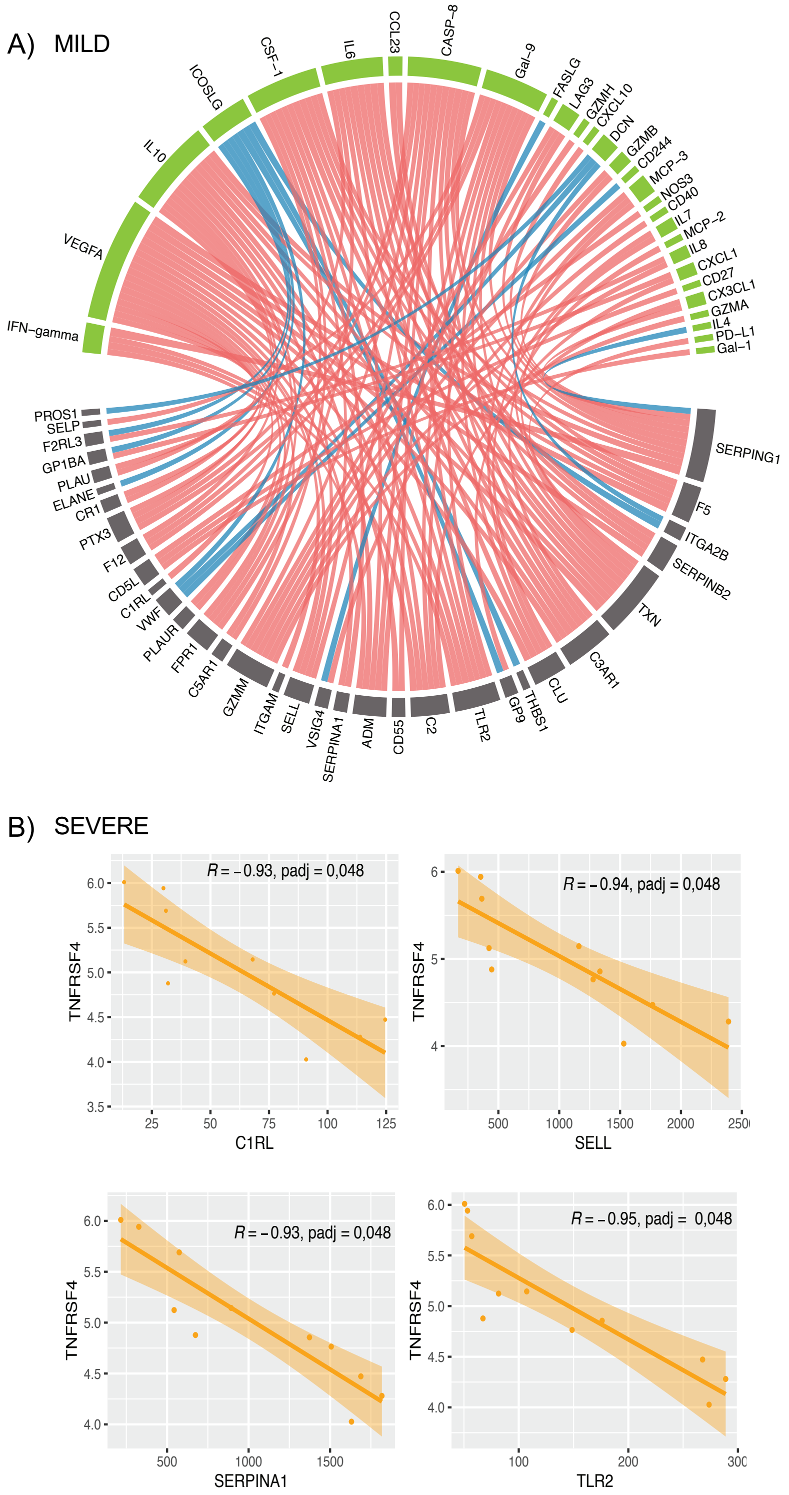

Supplementary Figure S4: Correlation between complement-coagulation gene expression and Olink plasma proteins in COVID-19. (A) Chord plot depicting significant correlations between complement-coagulation gene expression (grey bars) and plasma protein levels in NPX (green bars, Olink Immuno-Oncology panel) in mild COVID-19 patients. Red chords represent positive correlations, and blue chords represent negative correlations. Only correlations with adjusted p-value of <0.05 are shown. **(B)** Scatterplots of selected significant gene-protein correlations in severe COVID-19 patients, showing examples of robust negative associations such as TNFRSF4 (NPX; y-axis) with SELL, C1RL, SERPINA1, and TLR2 (TPM; x-axis). Each panel displays the correlation coefficient (R) and the adjusted p-value (padj). Correlations were computed using Spearman's method, and adjusted using Benjamini-Hochberg correction for multiple testing. An adjusted p-value of <0.05 was considered statistically significant.

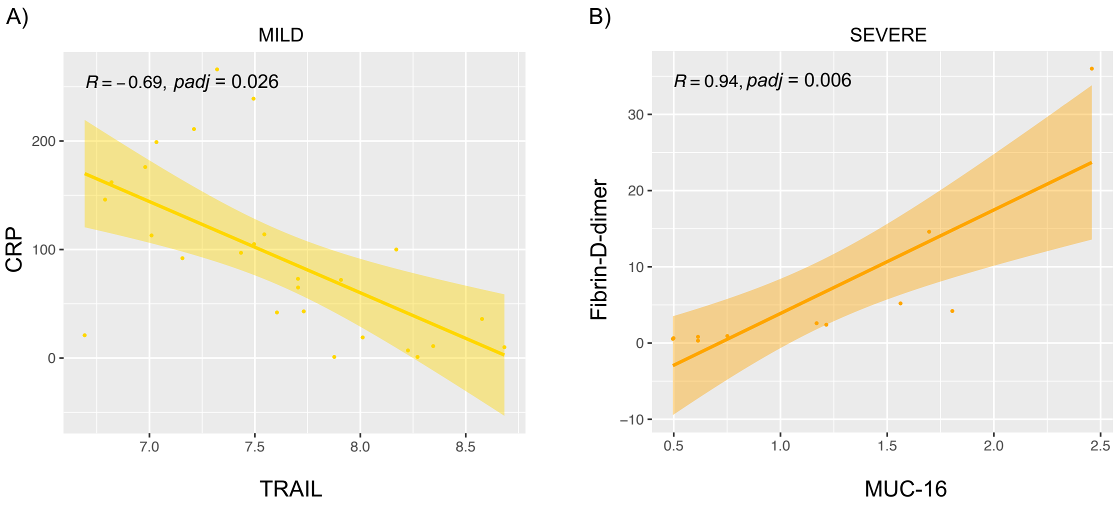

Supplementary Figure S5: Correlation between clinical laboratory parameters and Olink plasma proteins in COVID-19. Scatter plots illustrating significant correlations between clinical inflammatory/coagulation markers (y-axis) and Olink-measured plasma proteins (NPX; x-axis) in COVID-19 patient subgroups. **(A)** In mild COVID-19 cases, C-reactive protein (CRP; mg/L) levels show a significant inverse correlation with TRAIL (TNF-related apoptosis-inducing ligand) levels. **(B)** In severe COVID-19 cases, Fibrin-D-dimer (mg/L FEU) levels are positively correlated with plasma MUC16 (CA125) levels. Each panel displays the correlation coefficient (R) and the adjusted p-value (padj). All p-values were adjusted for multiple comparisons using the Benjamini-Hochberg method. Only correlations passing adj. p-value of <0.05 are shown.
